# Electroacupuncture alleviates orofacial allodynia and anxiety-like behaviors by regulating synaptic plasticity of the CA1 hippocampal region in a mouse model of trigeminal neuralgia

**DOI:** 10.3389/fnmol.2022.979483

**Published:** 2022-10-06

**Authors:** Yu-Zhi Jia, Hai-Tao Li, Guang-Ming Zhang, Hong-Yun Wu, Si-Shuo Zhang, Hong-Wei Zhi, Ya-Han Wang, Jing-Wen Zhu, Yi-Fan Wang, Xiang-Qing Xu, Cai-Jun Tian, Wen-Qiang Cui

**Affiliations:** ^1^First College of Clinical Medicine, Shandong University of Traditional Chinese Medicine, Jinan, China; ^2^Department of Neurology, Affiliated Hospital of Shandong University of Traditional Chinese Medicine, Jinan, China

**Keywords:** trigeminal neuralgia, anxiety, electroacupuncture, synaptic plasticity, hippocampal CA1 region

## Abstract

**Objective:**

Trigeminal neuralgia (TN), one of the most severe and debilitating chronic pain conditions, is often accompanied by mood disorders, such as anxiety and depression. Electroacupuncture (EA) is a characteristic therapy of Traditional Chinese Medicine with analgesic and anxiolytic effects. This study aimed to investigate whether EA ameliorates abnormal TN orofacial pain and anxiety-like behavior by altering synaptic plasticity in the hippocampus CA1.

**Materials and methods:**

A mouse infraorbital nerve transection model (pT-ION) of neuropathic pain was established, and EA or sham EA was used to treat ipsilateral acupuncture points (GV20-Baihui and ST7-Xiaguan). Golgi–Cox staining and transmission electron microscopy (TEM) were administrated to observe the changes of synaptic plasticity in the hippocampus CA1.

**Results:**

Stable and persistent orofacial allodynia and anxiety-like behaviors induced by pT-ION were related to changes in hippocampal synaptic plasticity. Golgi stainings showed a decrease in the density of dendritic spines, especially mushroom-type dendritic spines, in hippocampal CA1 neurons of pT-ION mice. TEM results showed that the density of synapses, membrane thickness of the postsynaptic density, and length of the synaptic active zone were decreased, whereas the width of the synaptic cleft was increased in pT-ION mice. EA attenuated pT-ION-induced orofacial allodynia and anxiety-like behaviors and effectively reversed the abnormal changes in dendritic spines and synapse of the hippocampal CA1 region.

**Conclusion:**

EA modulates synaptic plasticity of hippocampal CA1 neurons, thereby reducing abnormal orofacial pain and anxiety-like behavior. This provides evidence for a TN treatment strategy.

## Introduction

According to the International Headache Society and the third edition of the International Classification of Headache Disorders (Headache Classification Committee of the International Headache Society [IHS], 2018), trigeminal neuralgia (TN) is characterized by recurrent unilateral brief electric shock-like pains. It commonly occurs in the unilateral maxillary and mandibular branches of the trigeminal nerve and only occasionally in the ocular branches (Sindou and Brinzeu, 2020). TN is a chronic neuropathic pain which often causes mood disorders, such as anxiety and depression (Macianskyte et al., 2011). The negative emotions induced by chronic pain can further exacerbate the pain sensation (Bair et al., 2003). Thus, psychological therapies to improve mood disorders may be valuable adjuncts to pain management (Puskar and Droppa, 2015).

Electroacupuncture (EA) is a characteristic treatment in traditional Chinese medicine combining traditional acupuncture and modern electrotherapy. In recent years, EA has been widely used in the clinical treatment of TN and TN-related emotional disorders (Li et al., 2014; Cong et al., 2021). However, the analgesic and anxiolytic mechanisms of EA in TN have not yet been fully elucidated. The main function of Hippocampal is to receive complex comprehensive sensory and cognitive information, and it is one of the core regions processing pain, emotion, cognition, and memory signals (Roeske et al., 2021). Synaptic plasticity are closely related to these hippocampal functions. In chronic pain and abnormal emotional conditions, dysfunction in the CA1 region of the hippocampus, including fewer mature functional neurons, altered synaptic conformation and resting-state connectivity, abnormal expression of inflammatory cytokines and neurotrophic factors, reduced 5-hydroxytryptamine activity, and impaired glutamate transport metabolism, leads to impairments in synaptic plasticity (Khanna and Sinclair, 1989; Nakamura et al., 2010; Saffarpour and Nasirinezhad, 2021; Somelar et al., 2021). However, whether the alterations in hippocampal CA1 synaptic plasticity participate in the pathogenesis of pain and anxiety in TN is unknown.

In the current study, we used the mouse TN model of partially transecting the infraorbital nerve (pT-ION), which has been shown to induce stable pain and anxiety-like behaviors in our previous studies (Cui et al., 2020b). The ipsilateral acupoints (GV20 and ST7) were treated with EA or sham EA. The analgesic and anxiolytic effects of EA were evaluated using the von Frey test, acetone test, open-field test (OFT), and elevated plus-maze (EPM) test. Synaptic plasticity of the hippocampal CA1 region was observed by Golgi staining and TEM. The results showed that persistent orofacial allodynia and anxiety-like behaviors induced by pT-ION were related to changes in hippocampal CA1 synaptic plasticity. Repeated EA treatment improved pain- and anxiety-related behaviors and effectively reversed the abnormal changes in synaptic plasticity of the CA1 hippocampal region in pT-ION mice.

## Materials and methods

### Experimental animals

As experimental animals, adult male C57BL/6 mice (age 7–9 weeks, weight 22–25 g) were supplied by Beijing Vital River Laboratory Animal Technology Co., Ltd., Beijing, China. For acclimatization, mice were housed for 1°week before experiments under sterile and constant conditions (22 ± 1°C, 12-h:12-h diurnal cycle) with food and water *ad libitum*. The animal experimental procedures were performed in agreement with the standards of the Experimental Animal Management Committee of the Affiliated Hospital of Shandong University of Traditional Chinese Medicine and in agreement with the guidelines of the International Association for the Study of Pain. Extensive efforts were made to reduce the number of experimental animals and minimize their suffering in the experiments as much as possible.

### pT-ION model

**FIGURE 1:**
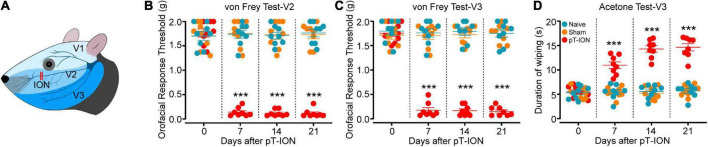
Time course of pT-ION-induced orofacial allodynia. Schematic diagram of orofacial region distribution innervated by trigeminal nerve and pT-ION surgery **(A)**. Primary mechanical hyperalgesia in the ipsilateral facial V2 area **(B)** and secondary mechanical hyperalgesia in the ipsilateral facial V3 area **(C)** induced by pT-ION are shown as reduced thresholds to mechanical stimulation. Secondary cold allodynia in the ipsilateral facial V3 area is shown as an increased wiping duration in reaction from acetone **(D)**. *N* = 8 mice/group, ***P < 0.001 vs. naive group, two-way ANOVA and Tukey’s test.

The pT-ION model was built as described in our previous studies (Cui et al., 2020a,b). Mice were anesthetized with intraperitoneal injection of pentobarbital sodium (50 mg/kg) and then placed in the supine position on the surgical pad, and the oral cavity was exposed. Then, an incision (0.5-cm long) was made in the left palatal-buccal mucosa, and the tissue and blood vessels were separated by blunt dissection to identify the ION of the trigeminal nerve. The ION was ligated with 4.0 catgut (BD171001, Boda Co., Ltd., Shandong, China), and the distal nerve was cut after 1–2 mm (Figure 1A). After this operation, the wound was sealed with tissue glue, and then the animals were kept in a warm chamber to recover from anesthesia. For the sham operation group, the left ION was exposed, but not ligated and clipped. Penicillin sodium (50,000 units/kg, dissolved in 0.9% normal saline, H20033934, North China Pharmaceutical Co., Ltd., Hebei, China) was administered intramuscularly to prevent wound infection for 3 days after pT-ION surgery. All operations were performed under sterile conditions. The naive group received no treatment. In all our experiments, only two pT-ION mice were excluded from subsequent experiments due to weight loss and behavioral abnormalities.

### Electroacupuncture treatment

The EA procedure used in this study is based on methods used in researches by us and our peers (Cui et al., 2019; Ulloa, 2021; Liu et al., 2022). From day 7 after the operation, mice in the pT-ION + EA group were treated with EA for 30 min once every other day for 14 days (i.e., on days 1, 3, 5, 7, 9, 11, and 13 for 30 min each) for 14 days. Mice were restrained in a special device so that their heads remained stationary while the limbs were free to move. An EA needle (0.18 × 13 mm, Suzhou Huatuo Medical Supplies Co., Ltd., Jiangsu, China) was used to puncture obliquely the Baihui (GV20, located in the middle of the parietal bone) and Xiaguan (ST7, located in the depression between the preauricular and mandibular notch) acupoints. For EA stimulation, the SDZ-II (Suzhou Huatuo Medical Products Factory Co., Ltd., Jiangsu, China) was used with the following stimulation parameters: EA frequency, 2 Hz continuous wave; current intensity, 1 mA (facial muscle twitch threshold). Mice of the pT-ION + sham EA group were treated with acupuncture needles inserted at the same acupoints (GV20 and ST7) for 30 min once every other day for 14 days, without administration of electricity.

### Behavioral studies

All behavioral experiments were performed at a fixed time in a quiet room with an appropriate temperature (22 ± 1°C) and soft light. To measure the mechanical and cold pain threshold, the von Frey test and acetone test were performed. The OFT and EPM tests were executed to assess anxiety-like behaviors of mice. All behavioral studies were performed by researchers blinded to the grouping of the animal.

### von Frey test

The von Frey test was conducted according to the method described by Dixon (Dixon, 1980). To reduce the effect of restraint on the behavioral evaluation of mice, the animals were placed in a box made of black silk screen (8 cm × 8 cm × 10 cm) and allowed to move freely. Before the test, the mice adapted to the test environment for two consecutive days (30 min a day). The oral mechanical stimulation thresholds of mice were determined before the surgery (day 0) and postoperative day 7, 14, and 21. We used a series of von Frey filaments (Stoelting, USA) with bending forces of 0.07, 0.16, 0.4, 0.6, 1.0, 1.4, and 2.0 g. Mechanical skin hypersensitivity of the ipsilateral V2 and V3 area of mice was tested by applying stimulation with increasing intensity starting from 0.07 g. To avoid interference, the hairs in the measurement area was ipsilaterally removed using a hair cutter (HC1066, Philips, Netherlands). Each filament was executed five times at 10-s intervals. The force of the von Frey filament with three-fifths positive reactions was recorded as the mechanical pain threshold.

### Acetone test

The cold pain threshold of the facial V3 area was evaluated by detecting the response to acetone-induced cold stimuli (Cui et al., 2020a). The mice were given 30 min to acclimate to the testing environment for three consecutive days before the test, and then a series of baseline measurements were carried out. Under mild restraint, the ipsilateral V3 skin of mice was treated with 50°μl 90% acetone (diluted with distilled water) using a modified syringe with a 25-gauge needle connected to a 1-ml microsyringe (Hamilton, Reno, NV). Experimenters taken sufficient care to prevent the acetone from affecting the eyes, mouth, and nose of the mice. The positive reactions were defined as the wiping of the ipsilateral V3 region by a front or hind paw (Cui et al., 2020b). The total orofacial wiping time was measured within a cutoff time of 2 min. An increase in wiping time after acetone exposure compared to baseline and sham group was defined as cold allodynia. It is difficult to avoid dropping acetone into the eyes when we administer acetone in the V2 region. Thus, primary cold allodynia of the V2 zone was not measured and only secondary cold allodynia of the V3 zone was tested.

### Open-field test

The OFT was administered as described previously (Jin et al., 2020; Luo et al., 2020; Chen et al., 2021). Under controlled conditions with constant temperature (22 ± 1°C) and dim light (15 lx), the mouse was placed at the center point of a square open field (50 cm × 50 cm × 40 cm) and allowed to explore freely for 5 min; the size of the center area was 25 cm^2^. Data of the free exploration movement were recorded and analyzed, including the total distances traveled, the time spent in the central area (central time) and number of crossing. After each test, we wiped the device with 75% alcohol and let it dry to prevent possible odor influences.

### Elevated plus-maze test

The EPM test was carried out as described in previous studies (Jin et al., 2020; Luo et al., 2020; Chen et al., 2021). The maze consisted of four arms (width, 5 cm; length, 30 cm) comprising two closed (black walls, 30 cm high) and two open (without walls) arms. The arms were connected by a central area of 6 cm^2^. The open and closed arms were made of dark plastic material, and the maze was lifted to a height of 50 cm above the ground by four plastic rods. At the beginning of the experiment, each mouse was released at the central point with its head facing the open arm and allowed to explore freely for 5 min. The time spent in the open arms (OA time) and number of entries in the open arms (number of OA entries) were recorded and analyzed. After the completion of the experiment, we wiped the maze with 75% alcohol and let it dry to prevent any odor influence.

### Rotarod test

To determine whether the locomotor function was altered in mice following EA treatment, the rotarod test was performed. Under controlled conditions with constant temperature (22 ± 1°C) and dim light (60 lx), the mice were placed on the rotarod (ENV-575M, Med Associates, USA) which was slowly accelerated from a speed of 4 rpm to 24 rpm in 180 s. The Rotarod CUB software was used to record the time before the mouse fell off the rotarod within this period. The test was repeated thrice for each mouse with 120-s breaks between trials, and the mean value was used for further analyses. On the day before the evaluation, the mouse was adaptively trained thrice with 30-min break in between. After each test, the rotating bar was wiped with 95% alcohol to eliminate odor effects.

### Golgi–Cox staining

Immediately after anesthesia, the brains of mice were removed, washed with 0.1 M PBS (4°C), and cut into three coronal segments. Following the instructions of a commercial Golgi staining kit (FD NeuroTechnologies, Inc., Columbia, MD, USA), the hippocampal tissue was removed and placed in a mixture of equal amounts of solution A and solution B, then stored in the dark at room temperature for 14 days. Afterward, the tissue was soaked in solution C and stored at 4°C for another 2°days. Hippocampal tissue sections (100 μm) were made under a cryomicroscope (HM 550; Thermo Science, Waltham, MA, USA) and placed in a mixture of solution D, solution E, and distilled water for 10 min. The sections were rinsed in distilled water and then dehydrated with various gradients of ethanol (50, 75, and 95%) for 5 min each, then rinsed with xylene and sealed with resin glue. After 24 h in the dark and under ventilated conditions, the slices were visualized using a microscope (Leica DM6000B, Germany). Images were reviewed, and the dendritic spine density was analyzed using ImageJ software.

### Transmission electron microscopy analysis

The brains were removed and placed in electron microscopy fixative (2% paraformaldehyde, 2% glutaraldehyde). The hippocampal tissue containing the CA1 section was cut in the fixative with a scalpel into a size of 1 mm^3^ and fully fixed at 4°C for 24 h. To avoid tissue contusion, care was taken to complete the extraction within 1–3 min and to minimize compression. After rinsing three times with 0.1 M PBS (pH 7.4), samples were fully fixed in 1% osmium tetroxide at 4°C for 2 h and dehydrated with different gradients of ethanol (30, 50, 70, 80, 95, 100, and 100%) and acetone (100%) concentrations. The samples were immersed in the immersion solution (acetone: EMBed 812 = 1:1, acetone: EMBed 812 = 1:2, pure EMBed 812) at 37°C. The pure EMBed 812 solution was poured into embedding plates, and the samples were embedded and kept overnight at 37°C. The embedding blocks were then polymerized at 60°C for 48 h and allowed to cool naturally into stiff tissue blocks. These tissue blocks were cut into ultra-thin sections of 100 nm and then stained in 2% uranium acetate, 6% lead citrate solution, and washed in ultrapure water. Subsequently, they were dried at room temperature overnight. The ultrastructure of the hippocampal CA1 region was observed using TEM (Hitachi Limited, Japan). At least 30 micrographs were selected per mouse, images were digitally acquired from 10 to 15 randomly selected areas, and synaptic density and structure analyses were performed using ImageJ software.

### Statistical analysis

All data of our study are presented as the mean ± SEM. GraphPad Prism 6 (GraphPad Software Inc., San Diego, CA, USA) was used to perform statistical analyses of the data. Two-way repeated-measures analysis of variance (ANOVA) followed by Tukey’s multiple comparison test was applied to analyze the time course of mechanical and cold allodynia. One-way ANOVA followed by Dunnett’s *post hoc* multiple comparison test or Tukey’s multiple comparison test was administered to analyze the statistical significance of differences between groups. *P* < 0.05 was considered the significance threshold in all analyses. The sample size was calculated based on our previous findings (Cui et al., 2020a,b).

## Results

### pT-ION induces unilateral orofacial nociceptive hypersensitivity and anxiety-like behavior in mice

The trigeminal branches include the ophthalmic nerve (zone V1), the maxillary nerve (zone V2), and the mandibular nerve (zone V3; Figure 1A; Basedau et al., 2022). It has been reported that the pT-ION model exhibits primary nociceptive hypersensitivity not only in zone V2 but also secondary nociceptive hypersensitivity in zone V3 (Cui et al., 2020b; Hu et al., 2020). The results showed no significant difference in basal pain between study groups before surgery, but the mechanical pain thresholds in the ipsilateral V2 and V3 zones of pT-ION mice decreased 7 days after surgery and stabilized until postoperative day 21 (von Frey test, V2: F_2_,_21_ = 138.3, *P* < 0.0001; von Frey test, V3: F_2_,_21_ = 204.1, *P* < 0.0001; two-way ANOVA and Tukey’s test; Figures 1B,C). The results of the acetone cold pain test in the V3 region showed that the duration of orofacial wiping in the V3 region of pT-ION mice significantly increased over the same time frame (F_2_,_21_ = 74.51, *P* < 0.0001; two-way ANOVA and Tukey’s test; Figure 1D), indicating that secondary cold pain hypersensitivity occurred in the pT-ION model. No significant differences in mechanical and cold pain thresholds were found between sham and naive groups from day 7 to day 21 after nerve injury. These results indicated that pT-ION can induce mechanical nociceptive hypersensitivity and cold allodynia in mice.

**FIGURE 2:**
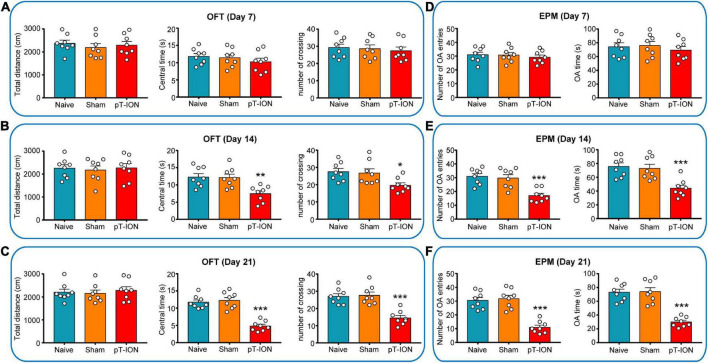
Open-field test (OFT) and elevated plus-maze (EPM) test results of mouse at various time points. In the OFT, the total distance, central time, and number of crossing were measured on days 7 **(A)**, 14 **(B)**, and 21 **(C)** after pT-ION surgery. In the EPM test, the number of entries in the open arms (number of OA entries), time spent in the open arms (OA time) were measured on days 7 **(D)**, 14 **(E)**, and 21 **(F)** after pT-ION surgery. *N* = 8 mice/group, *P < 0.05, **P < 0.01, and ***P < 0.001 vs. naive group, one-way ANOVA and Dunnett’s test.

To study whether and when pT-ION mice show anxiety-like behavior, the EPM and OFT test were performed on the 7th, 14th, and 21st days after nerve injury. The OFT results showed no significant differences on days 7, 14, and 21 after modeling between pT-ION and naive groups in total distance (day 7: *F* = 0.3372, *P* = 0.7176; day 14: *F* = 0.0879, *P* = 0.9162; day 21: *F* = 0.1984, *P* = 0.8216; one-way ANOVA and Dunnett’s test; Figures 2A–C). These results indicated that the locomotion ability of the mice was not affected by modeling. There were significant differences on days 14 and 21 after modeling between pT-ION and naive groups in central time (day 14: *F* = 8.293, *P* = 0.0022; day 21: *F* = 36.94, *P* < 0.0001; one-way ANOVA and Dunnett’s test), and number of crossing (day 14: *F* = 4.902, *P* = 0.0179; day 21: *F* = 19.77, *P* < 0.0001; one-way ANOVA and Dunnett’s test; Figures 2B,C), but not on day 7 (central time: *F* = 0.7437, *P* = 0.4875; number of crossing: *F* = 0.2017, *P* = 0.8189; one-way ANOVA and Dunnett’s test; Figure 2A). In the EPM test, the number of OA entries (day 14: *F* = 13.67, *P* = 0.0002; day 21: *F* = 30.51, *P* < 0.0001; one-way ANOVA and Dunnett’s test), OA time (day 14: *F* = 11.41, *P* = 0.0004; day 21: *F* = 31.39, *P* < 0.0001; one-way ANOVA and Dunnett’s test) of pT-ION mice was significantly decreased on days 14 and 21 after injury (Figures 2E,F), but not on day 7 (number of OA entries: F = 0.3295, *P* = 0.7229; OA time: F = 0.379, *P* = 0.6891; one-way ANOVA and Dunnett’s test; Figure 2D). These results suggested that pT-ION group show anxiety-like behaviors from day 14 to day 21 after nerve injury.

### Electroacupuncture attenuates orofacial allodynia and anxiety-like behaviors induced by pT-ION

**FIGURE 3:**
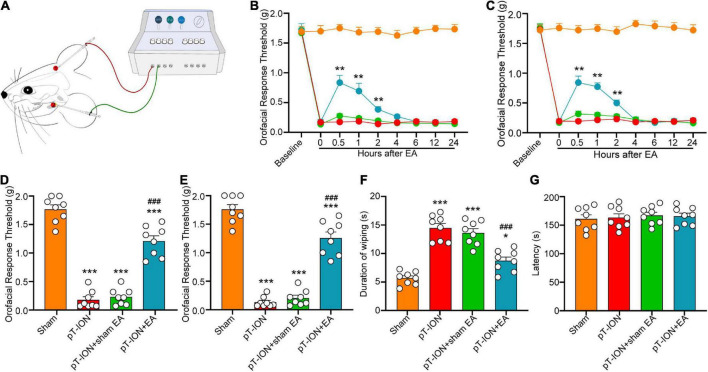
Electroacupuncture (EA) alleviates pT-ION-induced nociceptive abnormalities. **(A)** Schematic diagram of the EA procedure. EA acupuncture points (GV20 and ST7) are indicated by red points. Immediate analgesia caused by EA was measured after the first treatment on day 7 after modeling. Primary mechanical hyperalgesia was assessed in area V2 **(B)**, and secondary mechanical hyperalgesia was assessed in area V3 **(C)**, with peak analgesia 0.5 h after EA treatment. On day 21 after pT-ION surgery (day 14 after EA every 2 days), the analgesic effect of EA is shown as an increased primary mechanical pain threshold **(D)** and secondary mechanical pain threshold **(E)** in response to von Frey stimulation and a decreased wiping duration in response to acetone stimulation **(F)**. EA does not affect the latency to fall in the rotarod test **(G)**. *N* = 8 mice/group, *P < 0.05, **P < 0.01, and ***P < 0.001 vs. naive group; ^###^*P* < 0.001 vs. pT-ION group, **(B,C)** two-way ANOVA and Tukey’s test, **(D–G)** one-way ANOVA and Tukey’s test.

**FIGURE 4:**
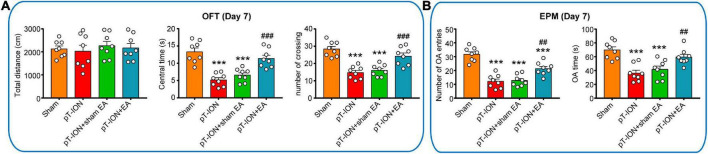
Electroacupuncture (EA) attenuates pT-ION-induced anxiety-like behavior. On day 21 after pT-ION surgery (day 14 after EA every 2 days), no effect of EA on motor function is shown as the constant total distance **(A)** in the open-field test (OFT). The anxiolytic effects of EA are shown as an increased center time and number of crossing in the OFT **(A)** and increased number of OA entries and OA time **(B)** in the EPM test. *N* = 8 mice/group, ***P < 0.001 vs. sham group; ^##^*P* < 0.01, ^###^*P* < 0.001 vs. pT-ION group, one-way ANOVA and Tukey’s test.

Electroacupuncture (EA) was applied on the acupoints GV20 and ST7 (Figure 3A). Immediate analgesia was observed after the first EA treatment on day 7 after modeling. EA significantly alleviated primary (F_3_,_28_ = 21.72, *P* < 0.0001; two-way ANOVA and Tukey’s test; Figure 3B) and secondary (F_3_,_28_ = 29.63, *P* < 0.0001; two-way ANOVA and Tukey’s test; Figure 3C) mechanical hyperalgesia induced by pT-ION at 0.5–2.0 h after treatment. These results indicated that EA has an immediate analgesic effect on pT-ION mice. On day 21 after nerve injury, the analgesic effect of EA was measured at 0.5–1.0 h after treatment. The mechanical pain thresholds in the V2 and V3 regions were significantly increased, and the orofacial wiping time was significantly decreased in the pT-ION + EA group compared to the pT-ION group (von Frey test, V2: *F* = 116.2, *P* < 0.0001; von Frey test, V3: *F* = 119.6, *P* < 0.0001; acetone test, V3: *F* = 40.53, *P* < 0.0001; one-way ANOVA and Tukey’s test; Figures 3D–F). To assess whether EA treatment impaired the locomotor function of mice, rotarod tests were performed at 0.5 h after EA, which was the time point that related to the maximal attenuation of orofacial hyperalgesia in EA-treated pT-ION mice. Both the fall time of rotarod test and the total distance of OF test showed no significant differences among groups (rotarod test: *F* = 0.1704, *P* = 0.9155; OF test: *F* = 0.2613, *P* = 0.8526; one-way ANOVA and Tukey’s test; Figures 3G, 4A). These findings demonstrated that EA has long-term analgesic effects on pT-ION model mice without disrupting their motor function.

To assess the anxiolytic effects of EA, the OFT and EPM test were performed 1–2 h after EA on day 21 after modeling. Compared with the pT-ION group, the central time, and number of crossing of the pT-ION + EA group were significantly increased, but the pT-ION + sham EA group had not significantly change in the OFT test (central time: *F* = 20.11, *P* < 0.0001; number of crossing: *F* = 19.85, *P* < 0.0001; one-way ANOVA and Tukey’s test; Figure 4A). In the EPM test, the number of OA entries and OA time were significantly increased in the pT-ION + EA group, but not in the pT-ION + sham EA group, compared with the pT-ION group (number of OA entries: *F* = 31.32, *P* < 0.0001; OA time: *F* = 12.53, *P* < 0.0001; open-arm entries percent: *F* = 31.51, *P* < 0.0001; one-way ANOVA and Tukey’s test; Figure 4B). As expected, EA significantly attenuated anxiety-like behavior induced by nerve injury, whereas sham EA had no significant effect.

### Electroacupuncture increases the density of total dendritic spines and mushroom-type dendritic spines in the CA1 hippocampal region of pT-ION mice

**FIGURE 5:**
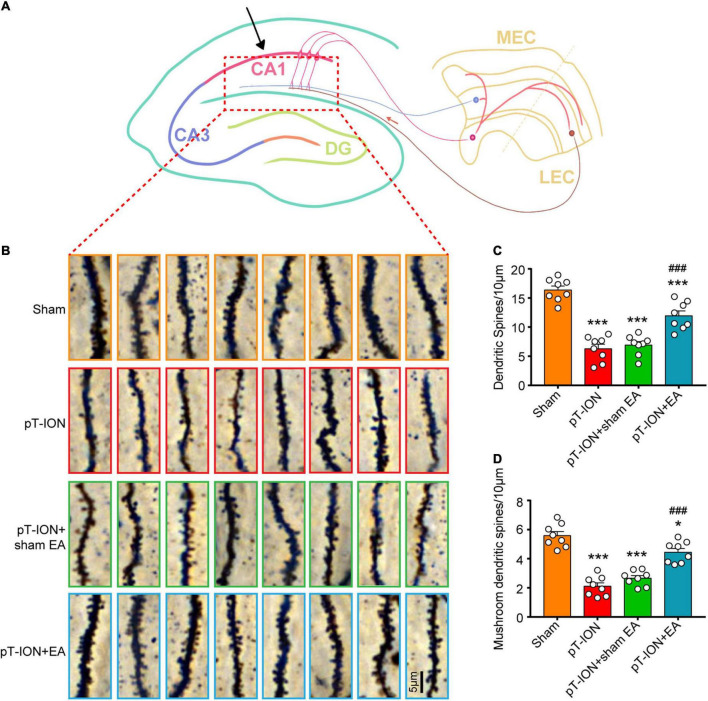
Electroacupuncture (EA) reverses the decrease in dendritic spine density induced by pT-ION in the CA1 hippocampal region of mouse. **(A)** Schematic diagram of the hippocampal region of mice. **(B)** Representative Golgi-Cox-staining images showing the density and morphology of dendritic spines in the cone cell layer of the murine hippocampal CA1 region in each group (scale bar: 5°μm). **(C)** Analysis of total dendritic spine density of the CA1 hippocampal region in each group. **(D)** Analysis of mushroom-type dendritic spine density in the CA1 hippocampal region in each group. *N* = 8 mice/group, *P < 0.05 and ***P < 0.001 vs. sham group; ^###^*P* < 0.001 vs. pT-ION group, one-way ANOVA and Tukey’s test.

Hippocampal structures, which mainly consist of the angular gyrus (CA1-CA3) and dentate gyrus, receive complex integrated sensory and cognitive information and are connected with the cortex (Figure 5A). To investigate the role of EA on synaptic plasticity, the dendritic spine density of the CA1 hippocampal area on day 14 after EA treatment was analyzed using Golgi staining. The total dendritic spine and mushroom-type dendritic spine densities were significantly decreased in the pT-ION group compared to the sham group (total dendritic spines: *F* = 42.38, *P* < 0.0001; mushroom-type dendritic spines: *F* = 43.32, *P* < 0.0001; one-way ANOVA and Tukey’s test; Figures 5B–D). EA treatment significantly increased the densities of total dendritic spines and mushroom-type dendritic spines in the CA1 region of pT-ION mice (Figures 5B–D). These results suggested that pT-ION damages dendritic spines and synaptic plasticity, and this effect can be reversed by EA.

### Electroacupuncture promotes the normalization of synapse number and structure in pT-ION mice

**FIGURE 6:**
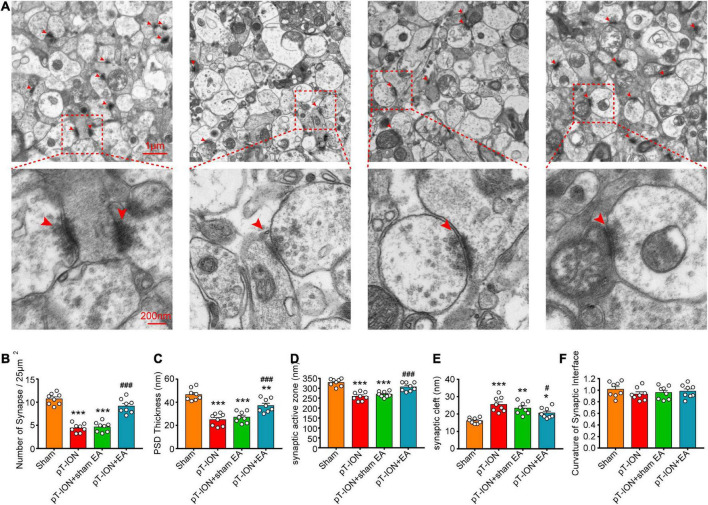
Protective electroacupuncture (EA) effects on density and ultrastructure of synapses in the CA1 hippocampal region of pT-ION mice. **(A)** Representative transmission electron microscopy (TEM) images showing the density and ultrastructure of synapses of the CA1 region in each group. The bar graphs show the number of synapses/25°μm^2^ **(B)**, the thickness of the postsynaptic densities (PSD) **(C)**, the length of the synaptic active zone **(D)**, the width of the synaptic cleft **(E)**, and the curvature of the synaptic interface **(F)** in each group. *N* = 8 mice/group, *P < 0.05, **P < 0.01, and ***P < 0.001 vs. sham group; ^#^*P* < 0.05 and ^###^*P* < 0.01 vs. pT-ION group, one-way ANOVA and Tukey’s test.

To study the effects of EA on synaptic plasticity, TEM was performed to observe the synaptic ultrastructure of the hippocampal CA1 region on day 14 after EA treatment. First, TEM results demonstrated that the synapses in the sham group had an arc-shaped asymmetric interface, with the presynaptic component containing many synaptic vesicles and active zones of approximately equal size, and the postsynaptic densities (PSD) of uniform thickness, separated by a clearly defined synaptic cleft (Figure 6A). Compared to the sham group, the number of hippocampal CA1 synapses (*F* = 55.3, *P* < 0.0001; one-way ANOVA and Tukey’s test; Figure 6B), PSD thickness (*F* = 28.56, *P* < 0.0001; one-way ANOVA and Tukey’s test; Figure 6C), and the length of the synaptic active zone (*F* = 23.99, *P* < 0.0001; one-way ANOVA and Tukey’s test; Figure 6D) were significantly decreased, and the synaptic cleft (*F* = 12.4, *P* < 0.0001; one-way ANOVA and Tukey’s test; Figure 6E) was significantly wider in the pT-ION group compared to the sham group. These results suggested that pT-ION causes a decrease in synaptic connections and synaptic transmission efficiency. Compared with the pT-ION group, the pT-ION + EA group showed a significant increase in synaptic density (Figure 6B), PSD thickness (Figure 6C), and length of the synaptic active zone (Figure 6D), as well as a trend in synaptic cleft narrowing (Figure 6E), whereas sham EA had no significant effect (Figures 6B–E). Moreover, there was no significant change in the curvature of the synaptic interface among groups (*F* = 0.7294, *P* = 0.5431; one-way ANOVA and Tukey’s test; Figure 6F). This suggested that pT-ION causes structural damage to synapses of the hippocampal CA1 region and EA can reverse this damage, improve synaptic plasticity, and increase synaptic transmission efficacy.

## Discussion

In animal studies, the orofacial allodynia induced by pT-ION are faster and more stable than traditional models of chronic constriction injury of the infraorbital nerve (CCI-ION) (Imamura et al., 1997; Ringkamp and Meyer, 2005). Thus, we used the pT-ION model by transecting the ION of mice in this study to mimic the onset of TN in humans. In clinical practice, the pain of traumatic TN (TTN) starts in the diseased branch. Over time, TTN pain may diffuse and spread across the entire facial dermatome (Benoliel and Gaul, 2017). Previous animal studies indicate that both chronic compression injury of the infraorbital nerve (CCI-ION) and pT-ION could induce primary (V2 skin) and secondary (V3 skin) allodynia (Shibuta et al., 2012; Kim et al., 2014; Guo et al., 2018; Hu et al., 2018; Cui et al., 2020a,b; Li et al., 2020). Consistent with the results of these studies, the primary and secondary mechanical as well as cold allodynia occured seven days after the pT-ION and persisted for at least 2°weeks in our study.

Chronic pain can cause emotional disturbances like anxiety, depression, and fear in affected patients (Hamasaki et al., 2018). Among chronic pain, TN is the most severe and debilitating (Smith et al., 2013; Zakrzewska et al., 2017). Thus, it adds to the probability of inducing mood disorders, including anxiety and depression (Obermueller et al., 2018; Jiang, 2019). Meanwhile, the phenomenon of TN inducing anxiety-like behaviors has been confirmed in the pT-ION and CCI-ION models (Alba-Delgado et al., 2013; Li et al., 2014; Cui et al., 2020b; Chen et al., 2021). Consistent with these studies, the current study also used the OF and EPM test to assess the anxiety-like behaviors of mice. Our results showed that the pT-ION model induced anxiety-like behaviors on days 14 and 21 but not on day 7, postoperatively. The parameters we analyzed included time spent in the central area and number of crossing in the OFT, time spent in the open arms and number of entries in the open arms in the EPM test. These parameters have been proved to have good validity in detecting anxiety-like behavior in animals (Jin et al., 2020; Luo et al., 2020; Chen et al., 2021).

Studies have demonstrated that EA has an analgesic effect on chronic pain and can modulate mood changes and cognitive dysfunction through central and peripheral mechanisms (Han, 2011; Chen et al., 2022; Zhou et al., 2022). In this study, EA had immediate alleviating effects on mechanical allodynia on postoperative day 7, and repeated EA alleviated the pT-ION-induced allodynia and anxiety-like behaviors on day 21 after nerve injury. Importantly, the mechanical pain threshold of the EA group on day 21 was higher than that on day 7, suggesting EA may have cumulative analgesic effects on pT-ION-induced orofacial pain.

Synaptic plasticity, a fundamental functional mechanism of neuroplasticity, has the ability to perceive, evaluate, and store complex information, as well as the ability to respond adaptively to relevant stimuli (Wang et al., 2020b). In chronic pain disorders, repetitive activation of pain circuits caused by injury, inflammation, and other factors induced the alterations in synaptic structure and function (Kuner, 2015; Tan, 2015). Behavioral symptoms of anxiety and depression are closely related to the decrease in hippocampal volume and synaptic loss (Noorani et al., 2022). Some previous studies demonstrated that EA attenuates neuropathic pain and depression by improving the synaptic plasticity of spinal cord and hippocampus (She et al., 2015; Chen et al., 2020; Zhou et al., 2022). However, whether EA alleviates orofacial pain and anxiety-like behaviors of TN by improving the synaptic plasticity of hippocampus is still unclear. The present study evaluated the anxiolytic effects of EA on TN-induced emotional disorders and explored its mechanism with respect to the hippocampal synaptic plasticity for the first time.

The alters of dendritic spine density and morphology are often used as a correlate of synaptic plasticity, and increased density is positively correlated with synaptic transmission (Wang et al., 2020a). The Changes of dendritic length and dendritic spine morphology were observed in hippocampal regions involved in pain information processing (Tyrtyshnaia and Manzhulo, 2020). Furthermore, dendritic spine loss and atrophy of hippocampal neurons may have a key role in the pathogenesis of depression (Duman and Duman, 2015). Our Golgi staining results showed that the densities of total and mushroom-style dendritic spines in the hippocampal CA1 region were significantly decreased at 21 days after pT-ION surgery, suggesting that synaptic plasticity in the hippocampus CA1 was disrupted. EA repaired the disrupted synaptic plasticity by increasing the densities of total and mushroom-style dendritic spines.

The alterations in the synaptic density, PSD thickness, length of the synaptic active zone, synaptic cleft, and synaptic curvature were considered to be one physiological base of synaptic functional plasticity that is closely related to synaptic function (Weeks et al., 2000). Pathological changes in synapses are frequently seen in neuropathic pain and depression, including an abnormal synaptic density, reduced PSD and associated proteins, shortened active zone length, decreased curvature of the synaptic interface, and widened synaptic cleft (Du et al., 2021; Yan et al., 2021). In our study, TEM analysis of synaptic ultrastructures showed that the number of synapses, PSD thickness, and length of the active zone in hippocampal CA1 were decreased in the pT-ION group, whereas the synaptic cleft was widened. EA increased the number of synapses, PSD thickness, and length of the synaptic active zone and narrowed the synaptic cleft. These results are consistent with findings of other studies on the mechanisms of drugs used to treat pain and depression (Du et al., 2021; Wang et al., 2022). Thus, the analgesic and anxiolytic effects of EA in TN may be mediated by the modulation of synaptic functional plasticity.

## Conclusion

This study demonstrates that EA has significant analgesic and anxiolytic effects on pT-ION-induced neuropathic pain and related anxiety-like behaviors. Our mechanistic studies indicate that synaptic plasticity of the hippocampal CA1 region, including the density, morphology, and structure of dendritic spines and synaptic, contribute to EA effects. Therefore, EA might be an effective treatment option, and the regulation of synaptic function and plasticity in the CA1 hippocampal region may be a potential therapeutic target for TN and TN-related affective disorders.

## Data availability statement

The original contributions presented in this study are included in the article, further inquiries can be directed to the corresponding authors.

## Ethics statement

The animal study was reviewed and approved by Institutional Animal Care and Use Committee of Shandong University of Traditional Chinese Medicine and Animal Ethics Committee of Affiliated Hospital of Shandong University of Traditional Chinese Medicine.

## Author contributions

W-QC, C-JT, X-QX, and Y-ZJ: study design/planning. Y-ZJ, H-TL, G-MZ, H-YW, S-SZ, H-WZ, Y-HW, J-WZ, and Y-FW: study conduct. W-QC: technical guidance. Y-ZJ, H-TL, and G-MZ: data analysis. Y-ZJ, H-TL, G-MZ, and H-YW: writing manuscript. All authors revising the manuscript, contributed to the article and approved the submitted version.
